# Application of NiTi in Assistive and Rehabilitation Devices: A Review

**DOI:** 10.3390/bioengineering6020037

**Published:** 2019-04-29

**Authors:** Mohammadreza Nematollahi, Keyvan Safaei Baghbaderani, Amirhesam Amerinatanzi, Hashem Zamanian, Mohammad Elahinia

**Affiliations:** 1 Dynamic and Smart Systems Laboratory, Mechanical Industrial and Manufacturing Engineering Department, University of Toledo, Toledo, OH 43606, USA; mnemato@rockets.utoledo.edu (M.N.); ksafaei@rockets.utoledo.edu (K.S.B.); 2 Department of Mechanical and Aerospace engineering, University of Texas at Arlington, Arlington, TX 76019, USA; amir.ameri@uta.edu; 3 Department of Bioengineering, Pennsylvania State University, CBEB Building, University Park, State College, PA 16802, USA; Hqz5213@psu.edu

**Keywords:** biomedical applications, NiTi, shape memory alloys, assistive and rehabilitation devices

## Abstract

Shape memory alloys (SMAs) have found widespread applications as biomedical devices. Biocompatibility, corrosion resistance, and ductility make these alloys attractive for medical devices such as stents and filters. For these implants, the superelastic property is the primary function of SMAs. Additionally, these alloys, such as NiTi as the prime example, can be used for actuation. Several modes of actuation such as displacement control, force control, and compliance control have been used as harnesses with SMA devices. These two unique properties have opened another application in the form of neurosurgery and robot-assisted surgery devices, as well as controlled assistive and rehabilitation devices. This paper reviews the state of the art of application of SMAs in the latter category where control is applied to harness innovative medical devices. To this end, two major subsets of these devices: prosthesis and orthosis which take the advantage of SMAs in assistive and rehabilitation devices are studied. These devices are further categorized to hand prosthetics, elbow, knee and ankle orthotics. In most of these designs, SMA wires act as artificial muscles to mimic the motion of limbs in the target joints. The evolution of each category is explained, and the specific results of them are reported. The paper also reviews the SMA applications for neurological and neuromuscular rehabilitation. To this end, different categories of rehabilitation devices as a passive and aided exercise for the ankle, knee, and elbow are highlighted. The SMA actuator in these devices can be EMG-controlled to improved patient outcome. In addition to providing a comprehensive overview of the biomedical devices, this paper identifies several possible future directions of SMA related research in the area of assistive and rehabilitation devices.

## 1. Introduction

Shape memory alloys (SMAs) are the metallic materials that have the ability to return to some previously defined shape when they are subjected to the appropriate thermo-mechanical loading. The shape memory effect occurs due to the temperature and the stress-dependent shift in the materials crystalline structure between two different phases called Austenite (parent) and Martensite (product). Martensite, the low-temperature phase, is relatively soft whereas Austenite, the high-temperature phase, is relatively stiff. This change in stiffness is a key enabler in realizing SMA assistive and rehabilitation devices. Changing in the SMAs crystalline structure is not a thermodynamically reversible process and results in temperature hysteresis [1]. The hysteresis provides shock absorption capacity which is essential in many applications.

SMAs are currently used in a wide variety of applications such as architecture [2], aerospace [3], automotive [4], and medical [5] fields. Of all existing shape memory alloys, Nickel-Titanium (NiTi) has proven to be the most beneficial in biomedical applications due to its biocompatibility as well as other unique properties. Properties such as biocompatibility, greater ductility, more recoverable motion, excellent corrosion resistance, and the ability to be electrically heated for shape recovery are the attractive features of NiTi [6]. NiTi SMA is the binary, equiatomic, intermetallic compound of nickel and titanium. In simpler words, it is approximately 50 at% Ni and 50 at% Ti. This intermetallic compound has a moderate solubility range for excess Ni or Ti, at a ductility comparable to most ordinary alloys. The solubility allows NiTi to be alloyed with other elements to change the mechanical properties and phase-transformation temperature. For example, adding additional Ni to the binary compound (up to 1% extra) strongly depresses the phase-transformation temperature and increases the yield strength of the Austenite. Iron and chromium can also be added to lower the transformation temperature. By varying the third element, the transformation temperature can be varied from −200 to 110 °C (−328 to 230 °F) [7]. Copper can be added to decrease the hysteresis and lower the deformation stress (de-twinning stress) of the Martensite. Generally, manufacturing NiTi SMA and shaping it for a specific purpose is not a simple task. Since Ti is a very reactive element, melting must be done in an inert atmosphere. Common methods are plasma-arc melting, electron-beam melting, and vacuum-induction melting. NiTi ingots can be initially shaped using standard hot-forming and cold-working processes. During cold-working, the alloy work hardens very quickly and must be annealed frequently. Work hardening and the correct heat treatment can be used to improve the SMA’s performance by reducing the stress needed to de-twin the Martensite and increasing the strength in the Austenite phase. Machining NiTi through cutting methods is difficult, as is welding, brazing, and soldering. Grinding, shearing, and punching are often better methods to create specific shapes. The “memory configuration” of an SMA part is defined by restraining the part in the desired shape, and then heat-treating at typically 500–800 °C (932–1472 °F) [7]. 

Still, the high ductility and high reactivity of this alloy make it hard to build parts with more complex shapes and geometries hence hindering many SMA applications as actuators. Additive manufacturing (AM) methods such as selective laser melting (SLM) and direct metal deposition (DMD) are the very recent breakthroughs in fabricating metallic parts which enable the researchers to produce complex, functional, and ready-to-use parts from a computer-aided design model [8]. Fortunately, over the past few years fabricating NiTi through AM methods has been of great interest. Fabrication [9], characterization [10], and heat treatment [11] studies are being carried out with the goal of tailoring the behavior of the as-fabricated parts.

Some key features which are discussed earlier, make the SMAs (NiTi alloy) an ideal choice in biomedical applications [12]. Therefore, designing biomedical devices by utilizing these alloys makes the new devices lightweight, silent to operate, and able to produce more force for motion. On the other hand, there are some limitations for the SMA including low efficiency of around 10%, low bandwidth, and difficulty in control due to the nonlinear behavior of SMA in both shape memory effect and superelastic modes. Another control issue is that the entire deflection of an SMA element occurs over a small temperature range resulting in challenging controllability of the actuators in partial contraction.

**Figure 1:**
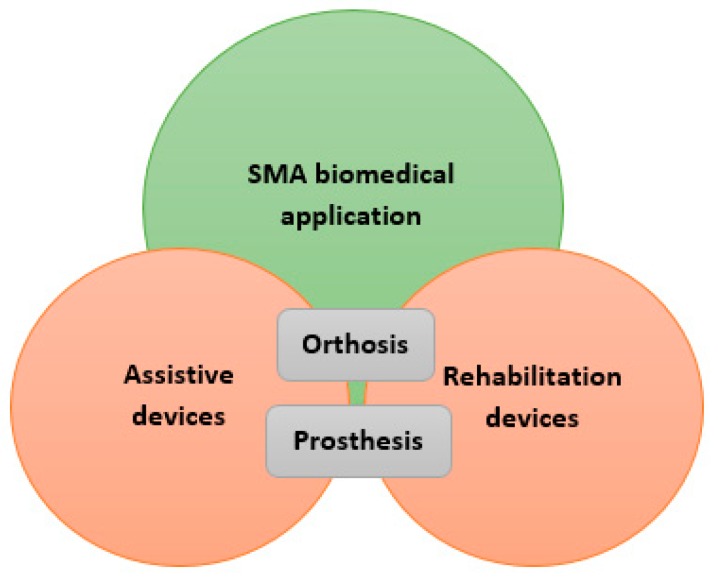
Schematic representation of sections studied in this paper.

According to the high variety of SMA applications in the biomedical domain, this paper studies the biomedical applications of the SMA (NiTi) in two main subsets: the assistive and the rehabilitation devices. The category shown in Figure 1 gives a clear overview of the topics discussed in the following. In each section, previous major works are presented, and their main approaches, technologies, and results are discussed. In the discussion section, the results of the all presented works are discussed and compared, and their main achievements are highlighted.

## 2. SMAs in Assistive Devices

### 2.1. Prosthesis

Prosthesis is an artificial substitute or replacement of a part of the body which enables people to do their daily activity normally. For example, lower limb prostheses enable a person with limb loss to walk, or a hand prosthesis helps the user to pick up things and lots of more functions that will eventually enhance the lifestyle of disabled people. As mentioned, the main problem of assistive devices such as prostheses is their actuation systems [13]. Consequently, researchers tried to take advantage of SMAs to overcome those drawbacks. For example, by using some NiTi wires, they can apply relatively large forces which results in much lighter devices compared to the devices used pneumatic, hydraulic, and electric actuation methods.

#### 2.1.1. Y. Nakano 

**Figure 2:**
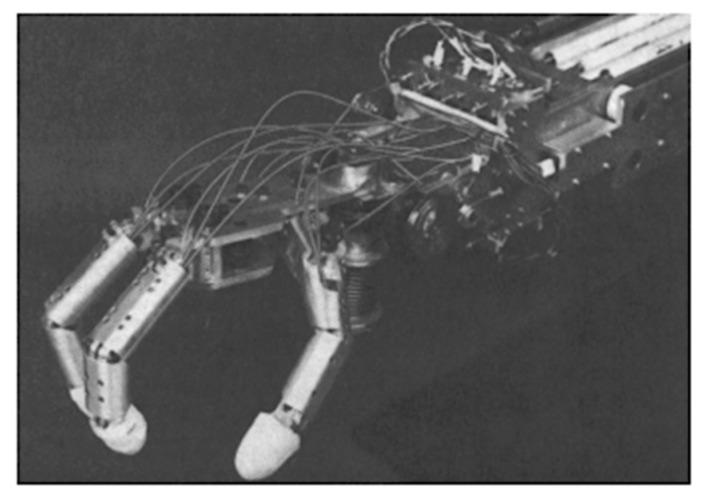
The Hitachi’s robot hand [14].

The Hitachi Hand was one of the first industrial hands and the most famous one that used SMA wires as artificial muscles [14]. Y. Nakano et al. developed the Hitachi Hand in 1984 and claimed a 10:1 reduction in weight in comparison to other hand designs. The four-fingered hand shown in Figure 2 includes the forearm and has 4.49 kg weight and 69.85 cm long. Joule heating caused SMA wires to be contracted against the spring force. SMA wires with 0.02 mm in diameter were used for the fingers and diameter of 0.035 mm ones set around a pulley, actuated each degree of freedom of the wrist. Potentiometers were used for joints position sensing of the mechanism above which had a rotation of 90 degrees.

#### 2.1.2. K. Laurentis and C. Mavroidis 

K. Laurentis and C. Mavroidis [15] imitated human hand anatomy to develop a five-fingered, twenty DOF, dexterous prosthetic hand. This device adopted shape memory alloy artificial muscles as actuators. Lightweight actuation (around 1.36 kg) was achieved through using a series of SMA wires which are noiseless and have a high power to weight ratio. Besides, a modular design for the attachment of the artificial muscles has been helped the users to just replace the muscles if they’re broken [16]. Finally, the first device was fabricated by using conventional methods and another by rapid prototyping techniques [17,18]. By taking advantage of the rapid fabrication method, the structure was built in one step without the need for any further assembly as well as having the property of lightweight, multi-finger, and multi-jointed structure [19]. Each joint of this device has a maximum rotation of 80°, and the range of motion for the rod-end joints for the four fingers is 90° in flexion and extension, and ±10° for lateral movement. Also, the thumb balls and socket joints have ±30° deflection in all directions.

#### 2.1.3. K. Andrianesis and A. Tzes

The anthropomorphic prosthetic hand developed by K. Andrianesis and A. Tzes [20,21] is an underactuated configuration of 16 joints with 7 degree of freedom (DOF) and targets to mimic the human anatomy as much as possible, in such a way that it has the shape and size of the average human hand (as each finger has one DOF and the thumb has 3 DOFs, so it is expected common daily activities to be easily performed. Another key feature of this design is using the tiny SMA wires of 200 μm in diameter which results in faster heat transfer and a lower time response of 2.2 s with respect to the thicker SMA wires or other shapes of SMA actuator such as rod, spring, and tube [22].

**Figure 3:**
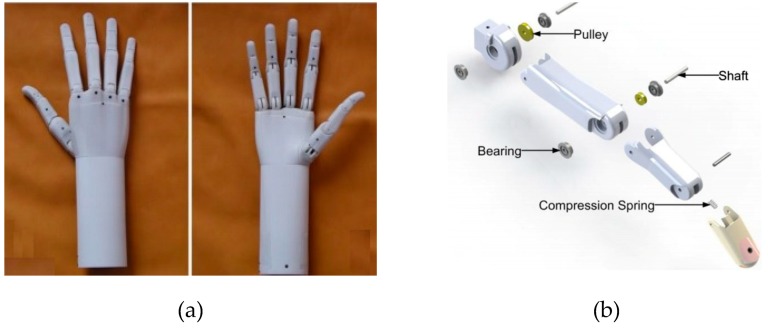
The humanoid prosthetic hand designed by K. Andrianesis and A. Tzes. (**a**) Prototype of the prosthetic hand (**b**) Exploded representation of a finger mechanism [21].

**Figure 4:**
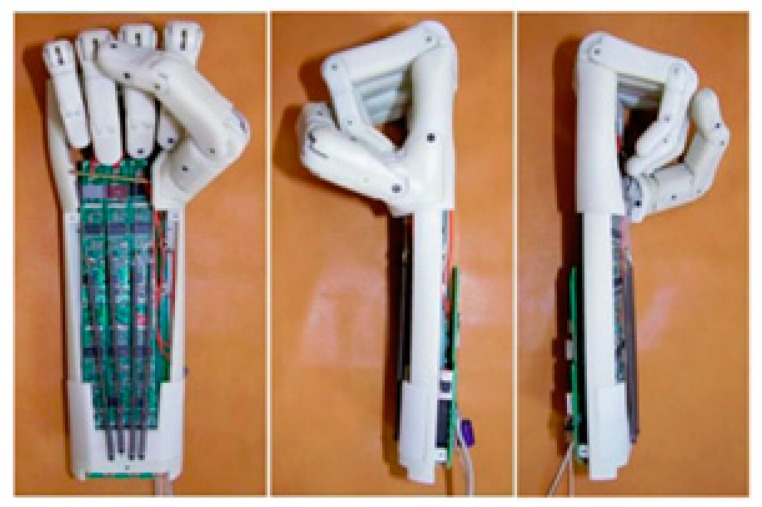
The SMA prosthetic hand took advantage of pre-tension spring and feedback control to have a better performance [21].

As a modification to this work, compression springs were added to the fingers to provide a powerless adaptive grip (Figure 3b). Further, a locking mechanism was designed to retain the fingers in the fixed positions when no actuation is provided to ensure powerless holding. As a modification to this work, pre-tensioned springs were added to the fingers to provide a powerless adaptive grip. Therefore, in the rest position, the hand is closed, and by applying electrical input, it will be opened (Figure 4). Also, these springs work as a bias force for SMAs to return them to the initial state.

Besides, the resistance feedback control was utilized for position controlling of the fingers. In their most recent work [21], force sensors also were employed to enhance the control performance during object grasping and enable sensory feedback. Capabilities of the designed hand were shown through experiments. Their final SMA actuated device had a 30% weight reduction (310 grams) in comparison to the current most advanced commercial prosthetic hands. 

**Figure 5:**
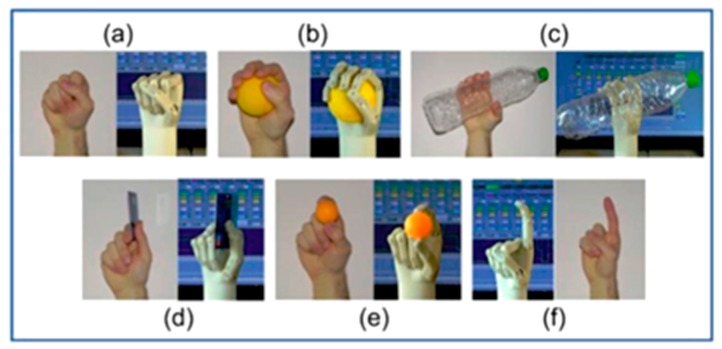
Different postures of a real human hand vs. the artificial hand [21].

Moreover, it had the advantages of low cost of SMA wires as actuators, decreasing the cost of additive manufacturing, using the self-sensing capabilities of the SMAs for controlling purposes, and typical usage of 50 W power [23]. Hence, taking advantage of all benefits mentioned before resulted in the humanoid prosthetic hand which imitates different postures of the real human hands as it’s shown in Figure 5.

#### 2.1.4. H. Taniguchi

**Figure 6:**
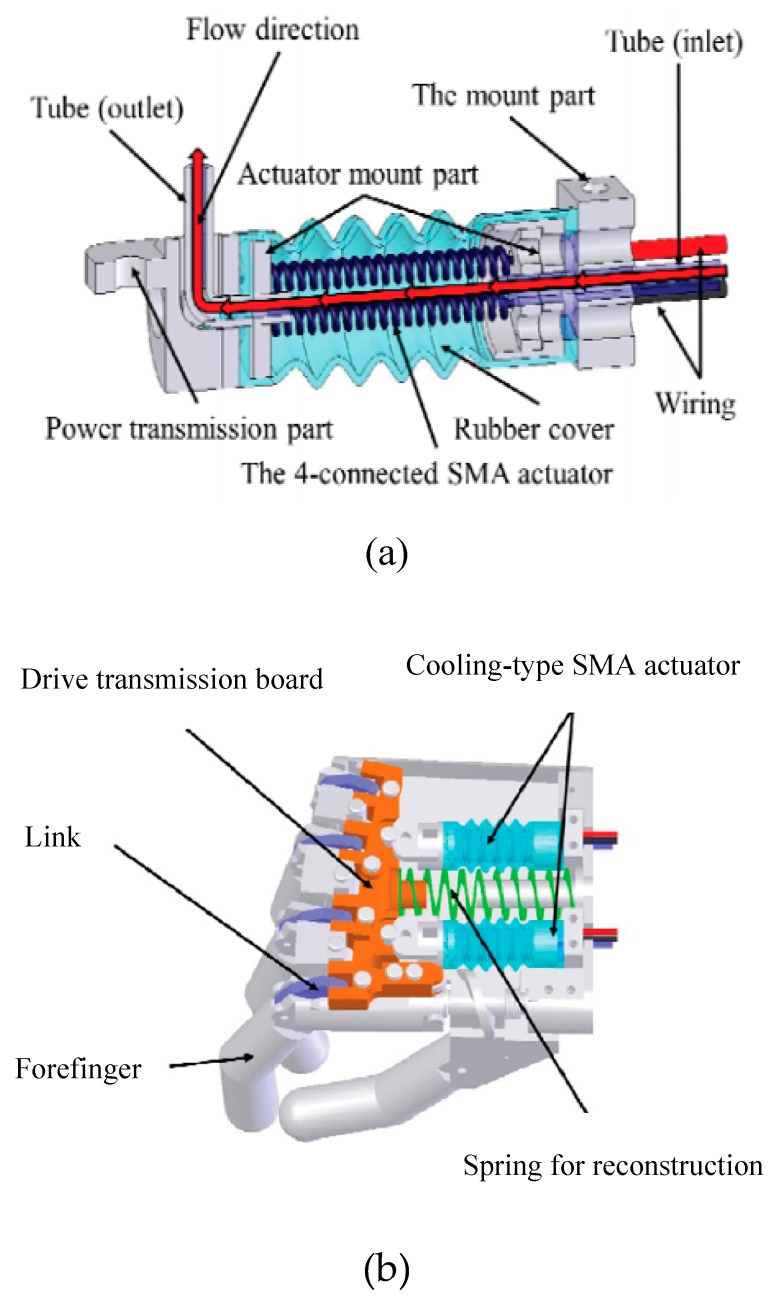
The prosthetic hand designed for children (**a**) Cooling system for achieving the higher actuation rate (**b**) the artificial hand structure [25].

H. Taniguchi designed a prosthetic hand for children by utilizing SMA actuators as the drive source to achieve a small size, silent operation, high output power, and low mass device [24,25]. To overcome the frequency limitation of SMA actuators, they used a forced cooling mechanism using a fluorine-based inert liquid with excellent electrical insulation and heat transfer properties as a coolant (Figure 6). It’s also experimentally shown that the prosthetic hand can apply a gripping force of 10 N which is suitable for children to hold dishes or change clothes.

#### 2.1.5. Jae H. Lee et al.

**Figure 7:**
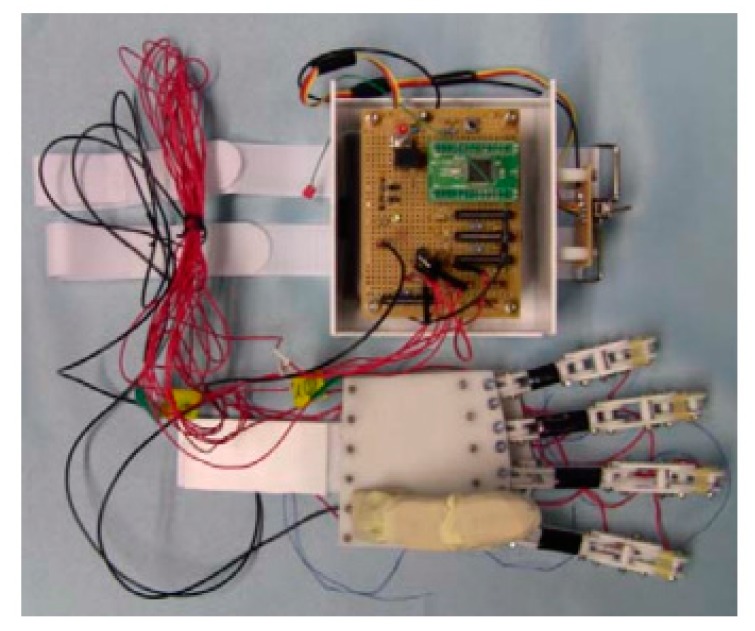
The prosthetic hand and the controller which is embedded into the palm [27].

Jae H. Lee et al. firstly designed a prosthetic finger based on the human hand structure and then developed it to the five-finger prosthetic hand [26,27]. Each of the first and the second fingers were actuated with two SMA wires, and the third and the fourth fingers were equipped with one SMA artificial muscle to support the other fingers, while the thumb is fixed to the palm. A microprocessor and a lithium cell have been located in the palm to control and drive each finger (Figure 7). Afterward, it was shown through some grasping experiments that the developed artificial hand is capable of grasping a variety of objects as well as holding lightweight ones.

### 2.2. Orthosis

Orthosis is an artificial external device providing support and making alignment for the movable parts of the body to correct deformities or to improve the functionality of the relative movement. Orthotic devices are designed in a way to assist the impaired limbs partially or totally lost their power. Having a high energy density, light weight, and stimulating by heating are some of the SMA properties that make these materials favorable for orthotic applications.

#### 2.2.1. SMA Orthotic Devices for Elbow 

##### Pittaccio et. al. 

**Figure 8:**
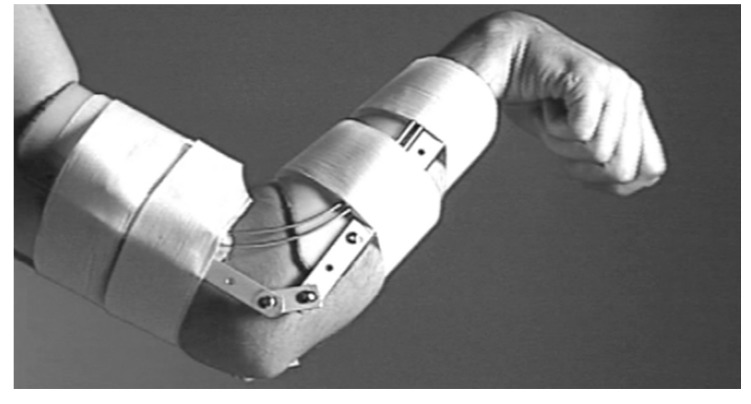
An EDGES orthosis prototype made up of two thermoplastic shells connected by a polycentric hinge at each side of the elbow joint [28,29].

Pittaccio et al. were among the first groups that investigated the potentiality of using superelastic NiTi in a compliant brace (EDGES) promoting spastic elbow relaxation [28]. Their hypothesis was that the unique pseudoelasticity character of NiTi could improve elbow posture without constraining movements and thus avoiding any pain to the patient. They used a commercial Ni50.7-Ti49.3 alloy heat-treated at 400 °C 1 h + WQ for their application. Then they assembled a prototype of EDGES with two thermoplastic shells connected by polycentric hinges. Four 2-mm diameter NiTi bars were encased in the upper-arm shell and let slide along tubular fixtures on the forearm (Figure 8).

**Figure 9:**
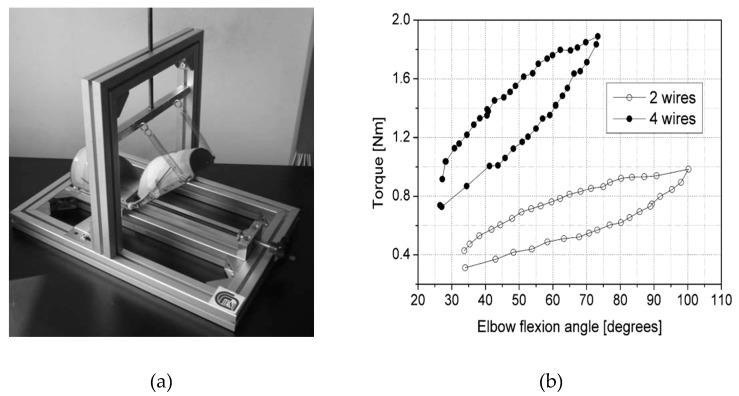
EDGES bending result, Data are shown by the dots [28]. An aluminum structure was designed for mounting the prototype and applying the torque (**a**); The results of the bending tests conducted on the assembled orthosis are presented in (**b**).

Several special designed bending tests were conducted to find an appropriate moment-angle characteristic. To show the torsion behavior of the wires in superelastic mode, an aluminum structure was designed for mounting the prototype and applying the torque (Figure 9a). Before mounting the wires, they are trained under a cyclic bending load condition to eliminate the residual strain. Two springs attached to the forearm are used to keep it straight and balance the moment caused by the wires bending. The results of the bending tests conducted on the assembled orthosis are presented in Figure 9b. The data were collected from several images which have been taken during the loading. The amount of torque was calculated from spring deflection and the angle measured from the images directly. The corrective torque versus angle characteristic of EDGES was nonlinear and hysteretic in both trials. Sufficient corrective push is also ensured at lower angles. The orthosis produces a moment increasing with an angle in the 20–80 range, after which it appears to reach a plateau at around 0.95 and 1.9 Nm when equipped with two and four wires, respectively. These values are considerably lower than maximal muscular flexor torques of around 50–75 Nm [29] and somewhat less than the maximal passive-reflex torques, which are reported to be in the range 0.5–6 Nm [30,31]. These measurements confirm that EDGES can stretch muscles while not constraining elbow position during voluntary movements or involuntary jerks.

Two post-stroke subjects wore EDGES for one week, at least 10 h per day. No additional treatment was applied during this period or the following week. An improvement (20° ± 5°) of the resting position was observed in both patients as early as 3 h after starting the treatment. As shown in Table 1, a slight decrease in spasticity was also observed in both subjects. All the effects disappeared one week after discontinuation.

**Figure 10:**
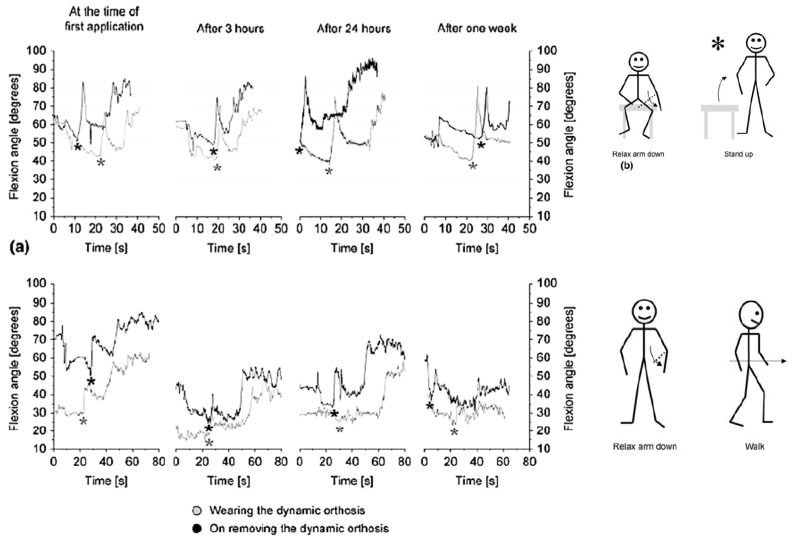
Clinical tests on EDGES. Optoelectronic acquisition of patients’ arm movement during the motion sequence: sit – relax arm down – stand up – relax arm down – start walking. The star marking the “stand up” command is followed by a sudden and pronounced flexion of the elbow (flexor synergies) [28].

The angle oscillation of flexion in Figure 10, expresses the fact that the EDGES orthosis applies the stable corrective force rather than a stable position to derive muscle stretching. In fact, this corrective force has been designed to produce muscle stretching without inhibiting arm movement. The clinical procedures carried out to assess the dynamic characteristics of EDGES when worn by spastic subjects showed that even in this un-optimized version it could produce corrective loads in a stable manner for at least a week and it does not constrain the elbow in a fixed position.

**Figure 11:**
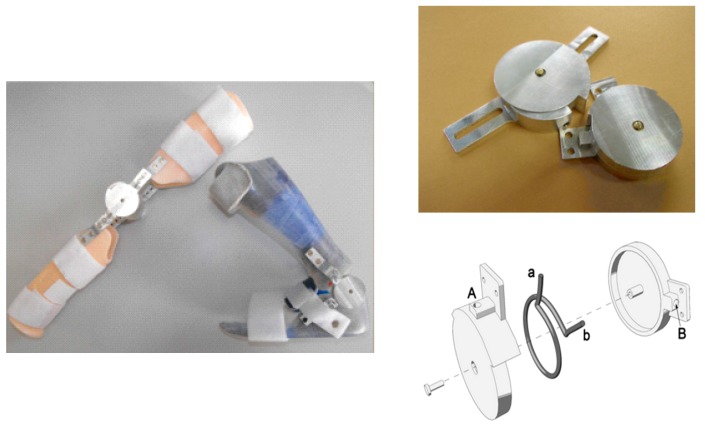
The pseudoelastic hinges consist of two parts rotating around a common central axis. Within the inner chamber formed by the two parts, a pseudoelastic Ω-shaped spring is inserted which generates a torque between two half-hinges. The ends (a and b) of the pre-loaded spring are inserted into the corresponding holes (A and B, respectively) in the parts [32,33].

In another study [32,33], Pittacio et al. designed and fabricated a specific hinge containing NiTi to transfer the pseudoelastic recovery force to fitted splints in the elbow joint. The pseudoelastic hinge presented in this paper (Figure 11) is composed of two parts which are able to rotate relative to one another around a common central axis.

In this study, the effect of thermal treatment on the superelastic alloys was considered to evaluate the changes on the torque-angle characteristics of the hinge. After evaluating the stability of the hinge for 20–100 cycles, the pseudoelastic hinge was used in a repositioning splint equipped, and the device was prescribed to the individuals with mild to severe spastic tetraparesis for the clinical evaluations (Table 2). The subjects’ age was between 4 and 18 with the Ashworth Score (AS) of more than 1 for the target limb. All subjects randomly divided into two groups, the first group wore the traditional static splint for one month and after that used pseudoelastic orthosis for the next month, and the second group used the orthosis in the opposite order. As the next step, the range of motion (ROM) and Ashworth score (AS) were measured for each patient.

Results on rigidity change for the sole elbow joints were not significant for either pseudoelastic treatment (p-value = 0.2234—paired 2-tailed Student’s T-test, n = 8) or traditional splints (p-value = 0.4367—paired 2-tailed Student’s T-test, n = 8). A potential reason for why differences between pre- and post-treatment values of rigidity were not significant for the elbow joints may depend on the size of the corresponding population. It’s not possible to make a reasonable decision based on the limited data obtained from only five candidates.

**Figure 12:**
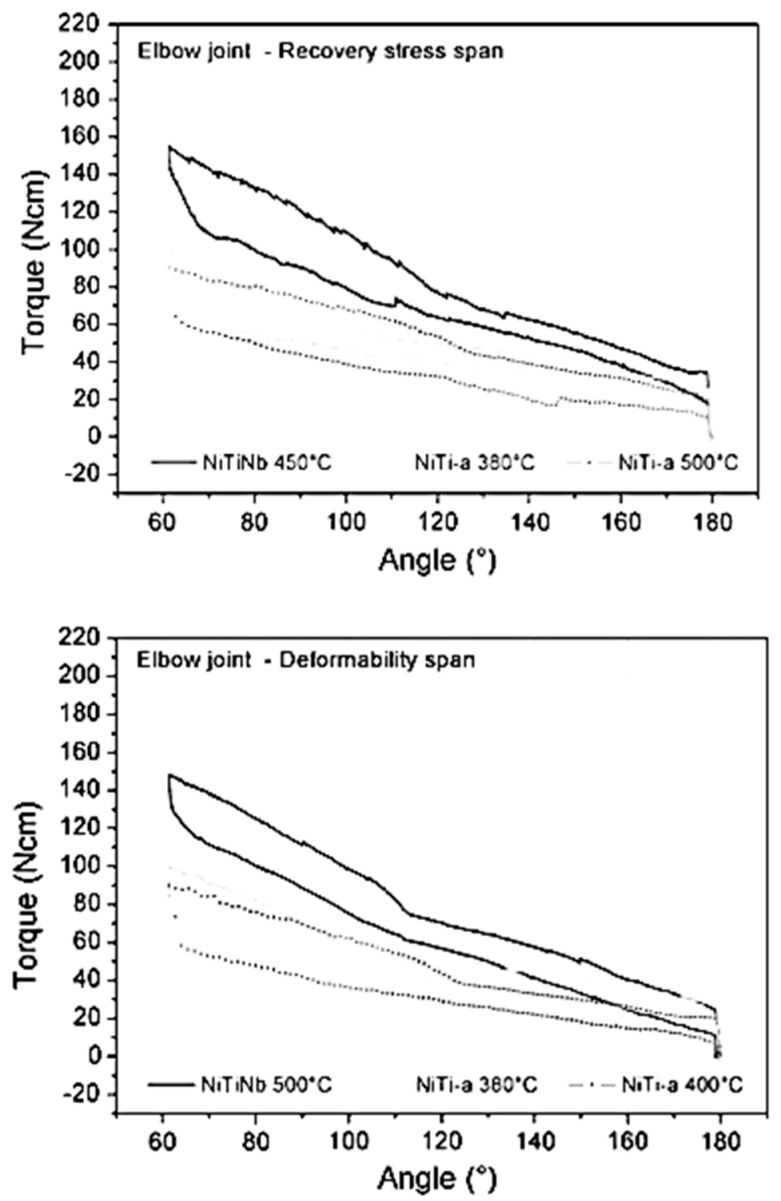
Torque–angle curves for the hinges mounting several different pseudoelastic springs. The graph above shows curves for a selection of hinges with pseudoelastic elements ranging across recovery stress levels; the graph below displays hinge properties for pseudoelastic elements spanning the plateau lengths [33].

By analyzing the curves of Figure 12, the trend in favor of the use of NiTi-a alloy for the elbow joint stands out, generally due to the flatness and length of the tensile unloading plateau. Because of the intrinsically larger pre-load imposed on the springs by the geometry of the ankle hinge, materials with softer characteristics (NiTi-c and NiTi-b) reach torque levels similar to those characteristics of NiTi-a in the elbow hinges. Conversely, for the same reason, NiTiNb-400 °C endows the ankle hinge with very high mechanical properties, whereas NiTiNb-500 °C applied to the elbow hinge (i.e., with less pre-load) produces a more moderate level of torques. On the whole, the prescribed orthoses provided different intensities of treatment ranging (in average terms across the allowed joint positions, considering the unloading plateau) from 25 to 150 N.cm per hinge (50–300 N.cm per orthosis). As a means of comparison, the maximal passive joint torques measured from the patients were in the range of 1000–2000 N.mm, with the upper end of the interval being typical of an elder subgroup aged 16–18 years. This means that the corrective torques applied were fairly mild. 

Based on the results obtained from the clinical evaluation of the patients, the pseudoelastic therapy appeared to outperform the traditional one in that it was able to conserve or improve joint viscoelastic characteristics, while the traditional treatment could not prevent stiffening.

##### Copaci et al.

**Figure 13:**
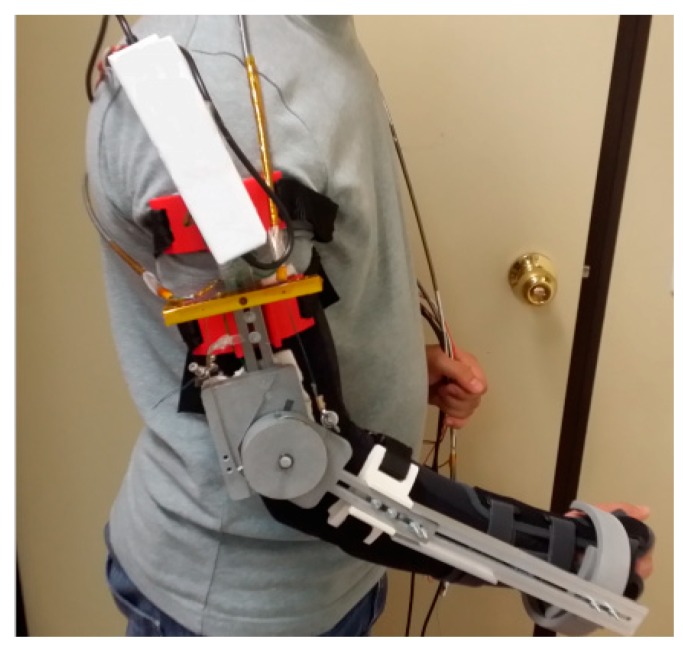
The SMA actuated exoskeleton with two DOF for flexion-extension and pronation-supination [35].

Copaci et al. designed a wearable elbow exoskeleton actuated with Shape Memory Alloy in the antagonist movement [34,35]. Their proposed elbow exoskeleton has 2 degrees of freedom (DOF) which can create flexion-extension and pronation-supination. An SMA based actuator has been designed to drive the elbow exoskeleton. Figure 13 shows the SMA actuated exoskeleton with two DOF for flexion-extension and pronation-supination.

In this study, the modified SMA based actuator consists of four wires of Shape Memory Alloy (SMA) inserted in one Bowden cable for the flexion movement and for the extension using the force of a torsion spring placed in the elbow exoskeleton joint. This modified configuration has a lower power consumption, offers a quick response, is more compact design, and can create more range of motion in comparison to the conventional devices which use DC and AC motor, hydraulic, and pneumatic actuators. The proposed elbow exoskeleton presents a lightweight, noiseless operation and can be built as a low-cost rehabilitation device by using a 3D printer and low-cost electronics and actuators.

##### Hope et al. 

**Figure 14:**
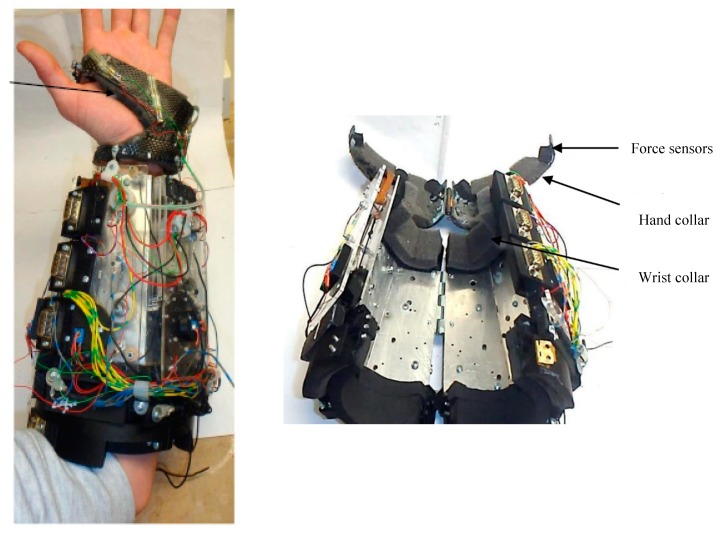
Three degrees of freedom prototype developed by Hope et al. for hand and forearm assistance [36].

They developed a wearable prototype with three degrees of freedom which facilitates robotic-assisted in-home rehabilitation therapy [36]. The prototype presented in Figure 14 consists of actuation modules which include SMA wires, force amplification mechanism, and position sensors. Modules have a differential configuration and include a bias spring. 

The controller of the system provides reasonable results in tracking sinusoidal trajectories. The theory of stiffness control in differential configuration was also implemented. Miniature fans were utilized to increase the cooling rate and decrease the response time of the system.

#### 2.2.2. SMA Orthotic Devices for Knee

Knee ankle foot orthoses (KAFOs) are prescribed to improve abnormal ambulation of the knee caused by quadriceps weakness. There are three major types of KAFOs: passive KAFOs, stance control KAFOs, and dynamic KAFOs. The passive KAFOs fix the knee joint in the full extension position all the time. Such devices provide enough support but cause stiff walking patterns. Some other types allow free knee motions inversely, producing limited stability [37]. Passive KAFOs are the most conventional type and are often prescribed for individuals with severe quadriceps weakness, broken bones, unilateral leg paralysis/paresis, and other similar diseases.

The stance control KAFOs prohibits knee movements in the stance while allowing free rotation at the knee in the swing [38]. They generate smoother gait compared to passive KAFOs. Also, gait compensation motions and energy consumption are reduced due to the flexible knee during the swing phase [39].

Dynamic KAFOs are the only type capable of controlling the knee motions throughout the entire walking gait cycle. However, those available in the market are heavy, bulky, and have limited functionality [40]. To address these issues, Feng et al. proposed using a superelastic Nitinol based dynamic knee actuating system for KAFO [41,42].

##### Feng et al.

**Figure 15:**
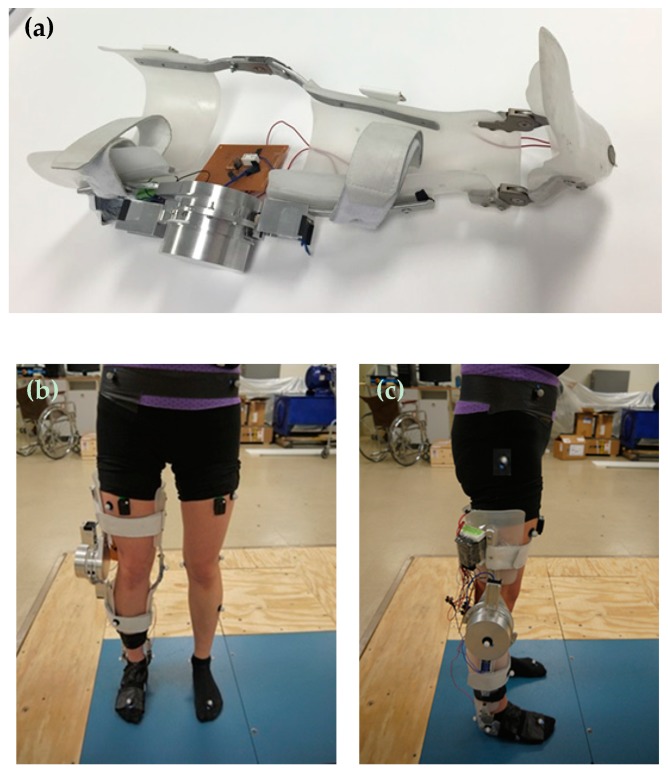
(**a**) The proposed KAFO Prototype with the Knee Module, the Control System, the Foot Switch, and the 12VDC Battery; (**b**) the frontal and (**c**) the lateral views of the subject wearing the dynamic KAFO for motion analysis test [46].

Feng et al. [43,44,45] were the first group that proposed using a superelastic Nitinol based dynamic knee actuating system for KAFO, which resulted in knee flexion and knee extension over the gait cycle, volume and weight reduction, and most importantly, a normal knee behavior. The developed a dynamic actuator consisted of two actuating parts, one working in the stance phase and another in the swing phases. Each actuating part was a combination between a superelastic torsional rod and a torsional spring in parallel. The surrounding parts of the dynamic knee joint were designed in a way to house both actuating parts. It is notable that the performance of the designed actuator had been verified prior to its fabrication by comparing the response of the actuator and the normal knee stiffness [46]. As seen in Figure 15a, the dynamic knee joint was fabricated and mounted on a conventional passive KAFO, replacing its original knee joint on the lateral side. Then, they evaluated the dynamic KAFO by conducting several motion analysis tests on a healthy subject while the subject is wearing the KAFO. Joint kinematics, kinetics, and electromyography data (detect quadriceps muscle activity) of both lower limbs were collected and calculated to compare the walking patterns in normal walking and walking with the proposed dynamic KAFO. Figure 15b,c demonstrate the subject is wearing the fabricated dynamic KAFO.

**Figure 16:**
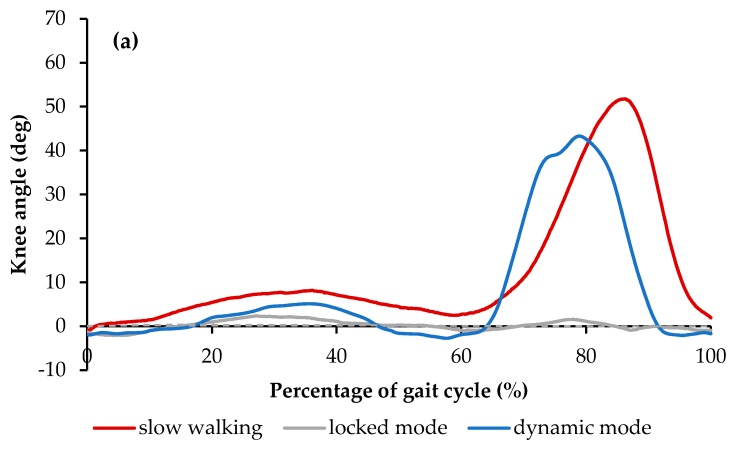

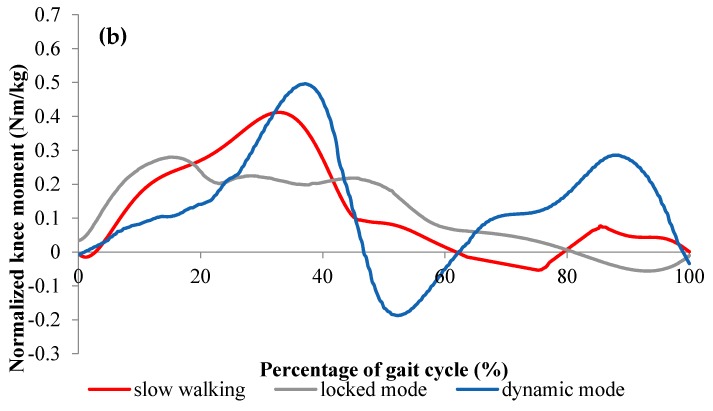
(**a**) Knee angle and (**b**) Knee moment over the entire gait cycle with one standard deviation in the three walking conditions [46].

Figure 16a demonstrates the results of the knee angle of the healthy subject, the subject walking with the conventional locked KAFO, and the subject wearing the dynamic KAFO. The data of the maximum knee angles on the orthotic leg during the stance phase were reported to be 9.3° ± 0.4° for the slow walking, 2.2° ± 0.3° for the locked mode, and 6.1° ± 0.5° for the dynamic mode. In the swing phase, the maximum knee angles were reported to be 60.6° ± 1.9° for the slow walking, 2.2° ± 0.3° for the locked mode, and 41.0 ± 4.3 for the dynamic mode. The reduction in the maximum knee flexion angle of the dynamic mode during the stance phase and swing phase were reported to be approximately 35% and 33%, when compared to those in the slow walking condition, respectively. However, the observed reduction in locked modes were 78% and 94%, respectively.

Figure 16b shows the weight-normalized knee moment profiles of the three conditions. The three graphs demonstrated a similar knee moment pattern over the gait cycle. When compared to the healthy subject, the dynamic mode reproduced higher and stiffer extension moments (up to 0.5 Nm/kg and 0.3 Nm/kg which are 25% and 200% more than the slow walking during stance and swing phases, respectively). Further, during the terminal stance phase, higher flexion moments (up to 0.2 Nm/kg which is about 150% higher than that in slow walking) were created in the dynamic condition. On the other hand, the locked mode demonstrated the lower extension moment when compared to the healthy subject (about 0.3 Nm/kg which is 25% less than the healthy walking).

**Figure 17:**
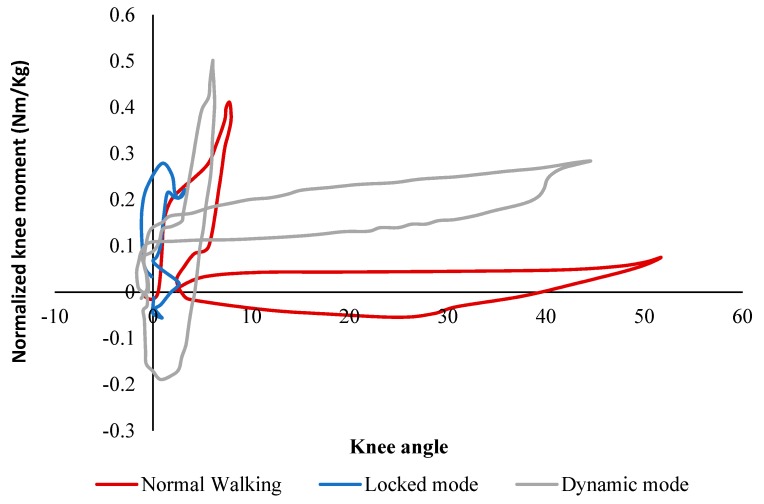
The Knee Stiffness Profiles (Weight-Normalized Knee Moment vs. Knee Angle) in the Slow Walking, the Locked Mode, and the Dynamic Mode [46].

For a better understanding, Figure 17 demonstrates the knee stiffness profile (calculated based on the measured joint angle vs. joint moment data) of the three conditions, which represent the knee moment versus the ankle angle over the gait cycle. In both slow walking and dynamic walking, the stiffness profile was composed of two distinct sections, one stiffer with less hysteretic behavior representing the stance phase, and another softer with more hysteresis representing the swing phase. However, in the locked mode, the stance phase was almost linear without hysteresis behavior without representing the swing phase. In slow walking, 0.4 Nm/kg internal extension moment and 9 degrees of flexion were observed in the stance phase and about 0.1 Nm/kg extension moment and 52 degrees of flexion were created in the swing phase. In the dynamic walking condition, approximately 0.5 Nm/kg with 6 degrees of rotation and 0.3 Nm/kg internal extension moments with 42 degrees of rotation was created in the stance and swing phases, respectively. On the other hand, in the locked walking mode, the maximum extension moment was around 0.3 Nm/kg, showing a different profile from the other two cases. The results indicate that slow walking and walking in the dynamic mode have close stiffness profiles. 

#### 2.2.3. SMA Orthotic Devices for Ankle

Ankle-foot orthoses (AFOs) are prescribed to assist the patients in achieving a more natural gait and to prevent muscle atrophy. Due to the high power to weight ratio, the SMA AFOs are lighter and have a simpler design in comparison to most of the conventional AFOs in the market which are more likely to be heavy, bulky, and to have a limited functionality. To address these issues, Bhadane et al. [47,48,49], Deberg et al., [50,51], Mataee et al. [52,53], and Amerinatanzi et al. [54] proposed using SMA based AFOs.

##### Bhadane-Deshpande et al.

**Figure 18:**
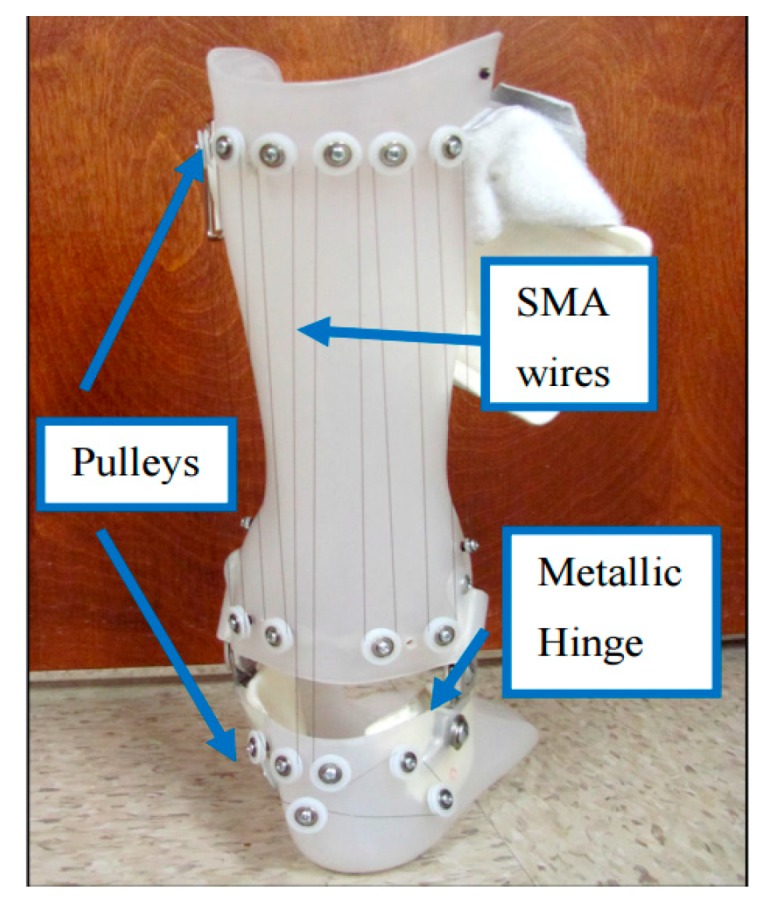
Pulley arrangement on hinged AFO to attach SMA wires [49].

Bhadane et al. proposed novel active AFO which used superelastic wires [47,48,49]. A total of fourteen plastic pulleys were attached on the brace using screws and spacers making a parallel combination of eight wires. The length of the SMA wire was calculated in a way to ensure complete strain recovery in one cycle. Considering the total angular variation of about 25 degrees, and constraining 4% strain recovery, the minimum length required for the wire was calculated to be about 90 inches. Figure 18 shows the setup of the developed AFO.

**Figure 19:**
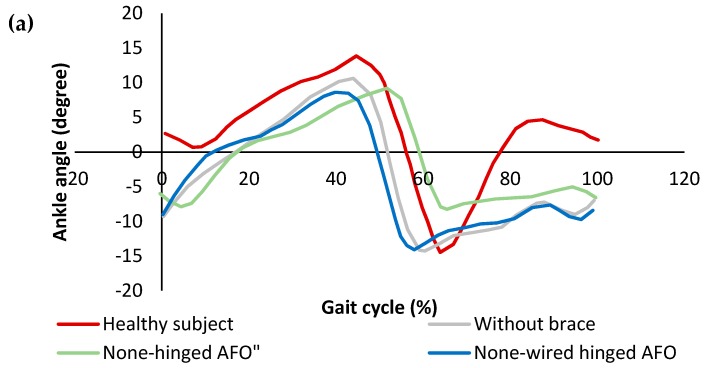

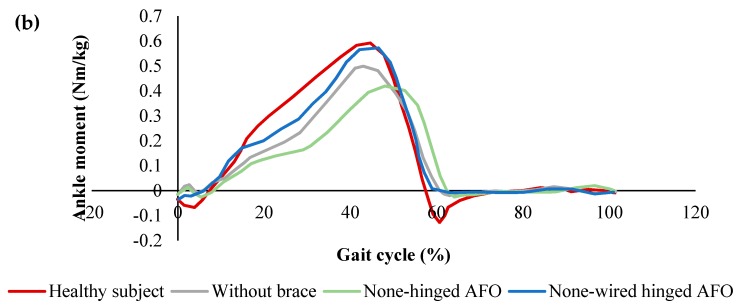
(**a**) Ankle angle variation, and (**b**) ankle moment variation over the gait cycle for the healthy subject drop foot patient with and without AFO [49].

Figure 19a shows the angular variation of the healthy subject, patient without AFO, with a non-hinged AFO (brace) and with a non-wired hinged (simple hinged system) AFO. The reason for this testing was to find the requirements for SMA wires which are considered to be added to the non-wired hinged AFO, such as the required level of angles and moments. It was seen that the drop foot patient was not able to dorsiflex his foot in the swing phase. A non-hinged AFO also was not able to help much in terms of assisting the foot to dorsiflex. Overall, it was inferred that the SMA wires should be added and provide the movement of the foot from 14-degree plantarflexion to 2-degree dorsiflexion. In Figure 19b, the angle moment variation was investigated for different conditions. The moment observed in both affected and unaffected leg of the patient was close to each other. The maximum deviation was observed in the late stance, where the muscles were not able to generate the push off. While non-hinged AFO increases the difference at the moment and restricts the ankle movement, the non-wired hinged AFO’s allowed for the movement and reduces the difference in the moment values and helps push off.

**Figure 20:**
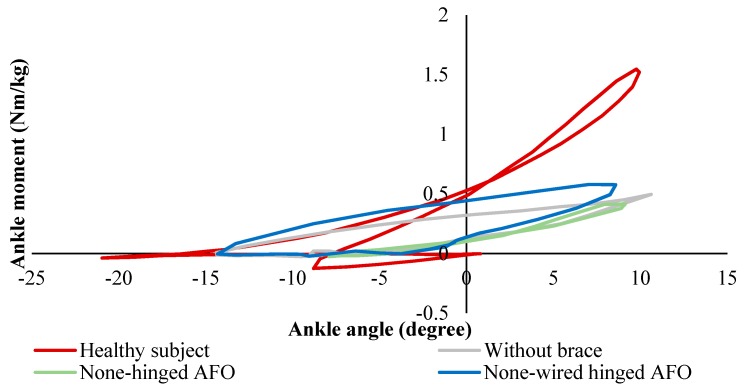
Ankle stiffness variation over the gait cycle for healthy subject and the drop foot patient with and without AFO [49].

In Figure 20, the stiffness profiles of all conditions were plotted to evaluate the stiffness characteristics required to be provided by SMA wires. While the non-wired hinged AFO provided more close stiffness to the healthy subject, the none-hinged significantly restricted the stiffness profile. To further improve the stiffness profile, it was proven that the additional stiffness was required to be added to the non-wired hinged SMA.

Comparing dynamic KAFO (Feng et al. design) and the SMA wire-driven AFO (Bhadane et al.) shows SMA components can be designed for KAFOs and AFOs to create more normal knee and ankle angle and moments to create a normal gate. As seen in Figure 18, Although Bhadane’s proposed AFO is not being controlled by an electronically powered SMA actuator, the length of SMA wire has been calculated in way that the strain recovery of the SMA wires assist the ankle joint for a more normal gate. 

##### Deberg et al.

**Figure 21:**
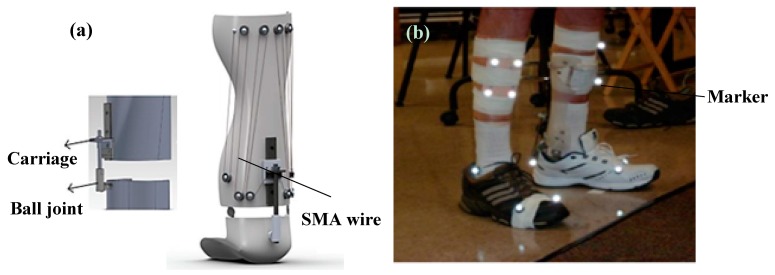
(**a**) The SMA passive AFO prototype, and (**b**) the motion analysis test on the patient wearing the SMA AFO [51].

Deberg et al. designed and evaluated a passive AFO utilizing the superelastic behavior of the SMA wires [50,51]. The SMA AFO presented in this work demonstrated the ability to meet the torque-angle requirements of the ankle assistive device much better than the typical brace or the conventional passive AFO. As it is shown in Figure 21a, the superelastic wires as the main elements of the device were fixed to the brace at one end and connected to the carriage at the other end. The carriage was considered to provide sufficient freedom by sliding on the slider connected to the ball joint. Several small pulleys were mounted on the brace to hold the required length of wire. The length of the wire not only affected the range of motion but also determined the lifetime of the orthosis. A longer wire could undergo less strain and therefore had a longer fatigue life. Figure 21b demonstrates the patient is wearing the SMA AFO for the motion analysis test. 

**Figure 22:**
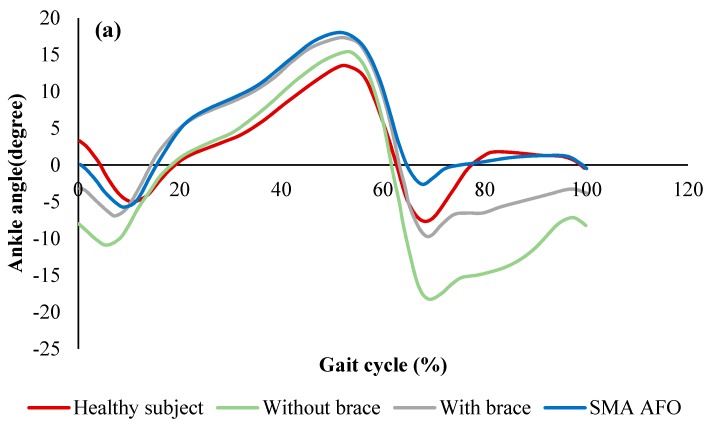

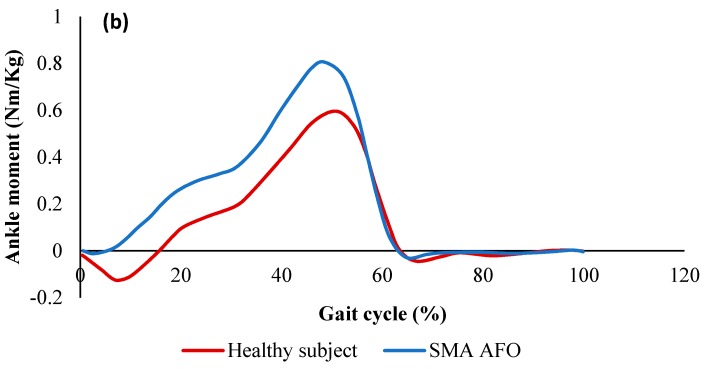
(**a**) Ankle angle (mean value) over the gait cycle for the healthy subject, the patient without the brace, with the conventional brace, and with SMA AFO, and (**b**) moment profiles over the gait cycle for the healthy subject and patient with SMA AFO [51].

Figure 22a shows the ankle angle of the healthy subject, drop foot patient without the brace, with a conventional hinged AFO, and with the proposed SMA AFO. When the patient was walking without the brace, a negative angle was observed at the end of the cycle which means the patient was not able to raise his foot in dorsiflexion phase. Although a more normal walking pattern was observed when the conventional brace was used, still negative ankle angle was observed in the dorsiflexion region. However, in the case of wearing SMA AFO, the patient was able to recover the ankle angle at the end of the phase. As shown in Figure 22b, the ankle moment was also comparable to the healthy subject when the patient was wearing SMA AFO.

##### Mataee et al.

**Figure 23:**
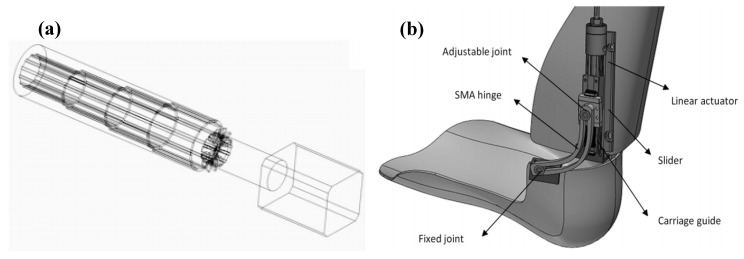
(**a**) Adjustable superelastic tube, and (**b**) adjustable superelastic hinge that provides the required level of stiffness in different walking conditions [53].

In another study, Mataee et al. presented two innovative SMA based adaptive solutions for the AFO mechanism based on the adjusting stiffness in bending and torsion [52,53]. The ultimate goal was to assist the patients in achieving a more natural gait and to prevent muscle atrophy. These concepts addressed the gait abnormality associated with drop foot patients for various walking conditions such as different walking speeds (slow, normal, and fast). In the first design concept in Figure 23a, a superelastic rod provided variable torsional stiffness via changing the inner diameter and length in response to different controlled axial loads. In the second design concept in Figure 23b, the active length of the superelastic hinge was adjusted by the position of the slider in order to control the bending stiffness. By adjusting the stiffness, the variable level of compliance was achieved at the ankle. In both concepts, energy was stored in the SMA during powered plantar-flexion in the stance phase of the gait. The release of this energy through superelasticity behavior enabled the AFO to provide the desirable dorsiflexion motion and to raise the foot during the swing phase of the gait. 

**Figure 24:**
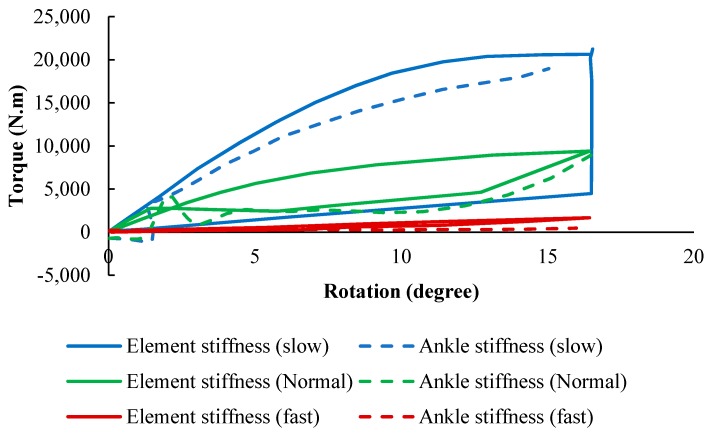
The stiffness results for SMA tube undergoing multiaxial loading (simulation) in comparison with that of the healthy ankle in normal, slow and fast walking [53].

For the adjustable superelastic tube, the ankle stiffness of the AFO (simulation) and that of the healthy subject was plotted in Figure 24 (experimental). A normal rotation was observed in the AFO due to the timely storing and releasing energy during the gait and the normal applied moment on the device. A softer behavior was achieved by increasing the portion of axial recovery and the pre-tension at the same time, which was desirable for faster walking speeds. Also, a normal behavior was observed by increasing the portion of axial recovery and decreasing the pre-tension, which was desirable for normal walking. Finally, a stiffer behavior was observed by decreasing the portion of axial recovery and pre-tension, which was desirable for slow walking. 

**Figure 25:**
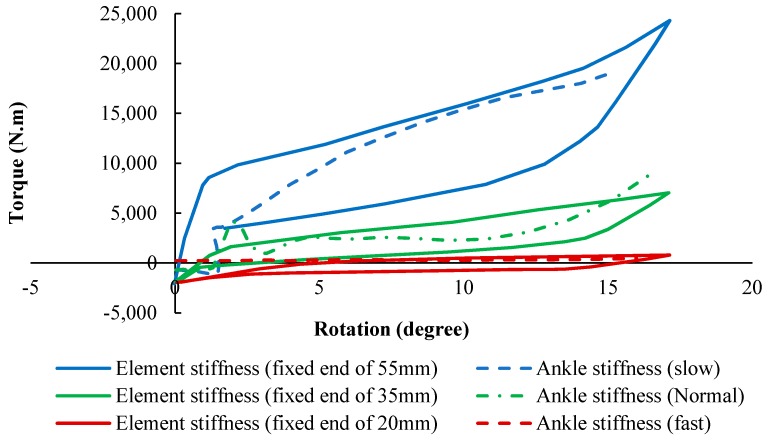
The stiffness results for SMA hinge fixed in different positions (simulation) in comparison with that of the healthy ankle in normal, slow and fast walking [53].

Similarly, for the adjustable hinge concept, the stiffness profiles of the AFO (simulation) and that of the healthy subject were plotted in Figure 25 (experimental). For three different hinge lengths which were set via the linear actuator position, the dimensions of the hinge were optimized to provide the desired stiffness profile for these three various speeds of walking. To follow the ankle stiffness in slow walking, the length of the hinge was fixed at the end of 55 mm, so that the element exhibited stiff behavior. To mimic the stiffness profile of normal walking, it was fixed to a length of 35 mm. Finally, the element was fixed at 20 mm to show a soft behavior and mimic fast speeding. The results indicated that the lower stiffness curves cover fast gait speeds occurring within the higher percentile of element length, and the higher stiffness curves cover slow gait speeds occurring within the lower percentile of element length. 

##### Amerinatanzi et al.

**Figure 26:**
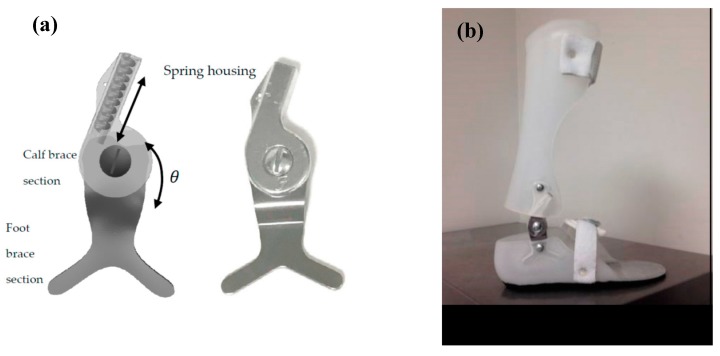
(**a**) Designed superelastic hinge AFO and (**b**) AFO porotype [54].

In another study, Amerinatanzi et al. developed an AFO that helps patients to have more normal ankle joint behavior [54]. The proposed AFO device consisted of a two-part brace, two superelastic SMA springs, and two hinges with a hole inside for mounting the springs (Figure 26).

**Figure 27:**
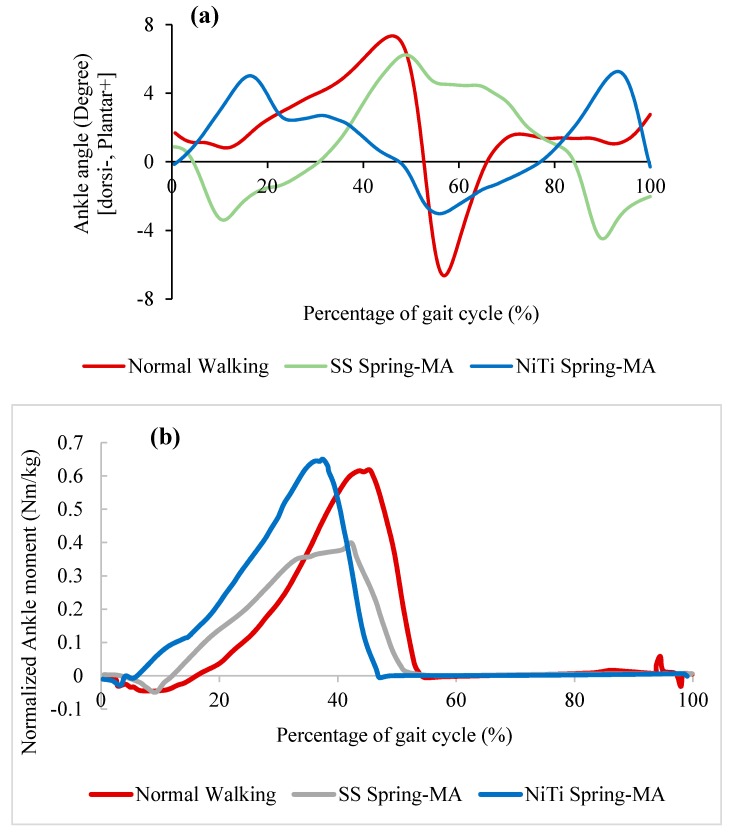
(**a**) Ankle angle and (**b**) normalized ankle moment over gait cycle for the healthy subject, subject wearing AFO with stainless-steel spring), and subject is wearing AFO with superelastic NiTi spring. (MA: Motion analyzed data) [54].

Figure 27a demonstrate the ankle angle over the gait cycle obtained from motion analysis for three different conditions: Healthy subject, the patient wearing conventional stainless-steel spring based AFO, and patient wearing superelastic NiTi spring based AFO. It was observed that the implementation of stainless-steel spring limited the ankle angle motion from 1-degree plantarflexion to 2.6-degree dorsiflexion. However, in case of using superelastic NiTi springs, the ankle angle motion was ranged from 5-degree plantarflexion to 3-degree dorsiflexion. Therefore, it was concluded that a more normal range of motion was observed when NiTi springs were used. Also, the patient was able to raise his/her foot to dorsiflex at the end of swing phase using NiTi spring based AFO, while plantarflex was observed at the end of the gate for the case of using stainless-steel spring based AFO. Figure 27b demonstrates the moment variation over the gait cycle for similar cases. The deviation from the normal moment was decreased from 0.24 Nm/kg to 0.05 Nm/kg when the stainless-steel springs were replaced with superelastic NiTi springs.

**Figure 28:**
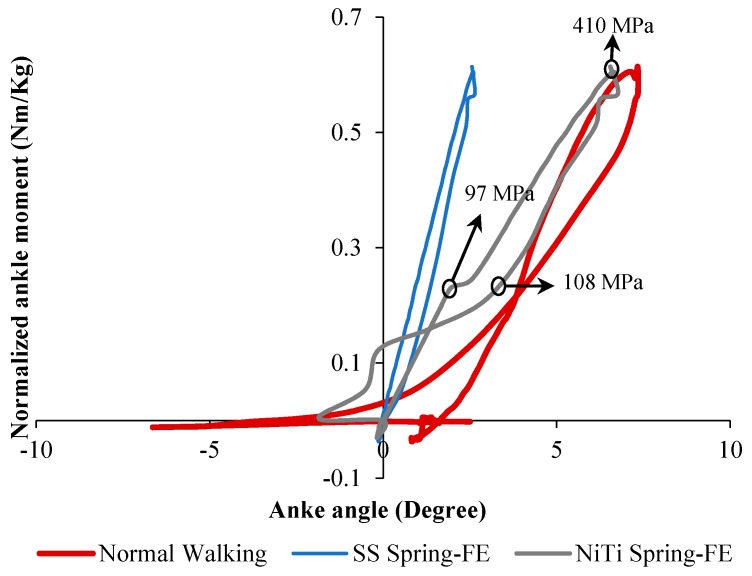
Ankle stiffness variation of drop foot patient with and without AFO [54].

Figure 28 shows the stiffness profile obtained from finite element analysis for three different conditions: Healthy subject, the patient wearing conventional stainless-steel spring based AFO, and patient wearing superelastic NiTi spring based AFO. In the case of using NiTi spring, the ankle showed a closer stiffness profile to the normal walking. However, in the case of using stainless-steel springs, the ankle stiffness was almost linear, showing a large deviation from the normal subject.

#### 2.2.4. Orthosis for Correcting Deformities and Abnormal Postures

The misalignment of the bones of the big toe is one of the reasons for creating the abnormal gate. The deformity may be in the digits of the hand, foot or other parts like the spinal skeleton. To solve this problem, some braces have been utilized to be fastened to the abnormal part to correct the abnormality [55]. The treatment for such problems requires almost a long time and may cause pain for the user.

**Figure 29:**
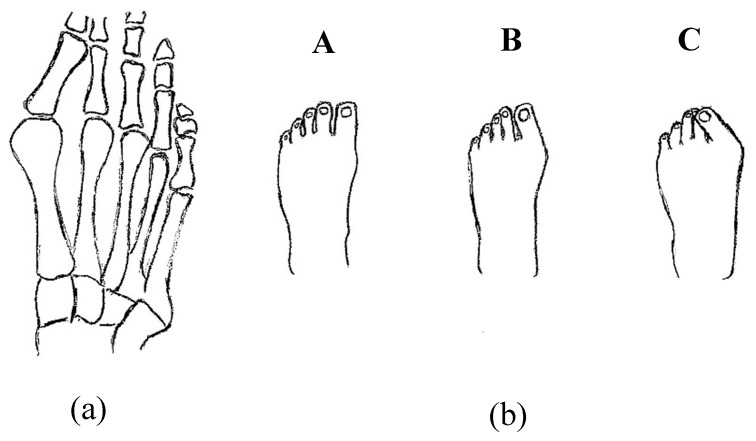
Toe deformities (**a**) bone positions and (**b**) the different level of deformities which deteriorate from normal toe position (A) to (B) to the worst posture (C) [56].

Palmer et al. invented a novel superelastic NiTi orthosis that maximizes the ability to slowly correct toe deformities (as presented in Figure 29) using the viscoelastic properties of the bone. The superelastic NiTi can gradually correct digit curves and ultimately results in better toe alignments compared to contemporary static splints or surgery. The disclosure comprises the provision and use of a splint that incorporates a shape memory material that is designed to generate and maintain a force that counteracts the direction of the bone deformity [56].

Implanted shape memory materials such as Nitinol can be utilized for assisting the spine motion. Wong and Nielsen invented a plate for sagittal correction of the spine. They developed a scoliosis mono-piece plate, to be fitted tightly on the back of a user. It comprises a lower section in addition to an upper section. The upper section is rounded in the sagittal plane, provided that the plate forces the thoracic spine of the user to bend forward in the sagittal plane [57]. In addition, Cheung et al. managed a five year study on a randomized double-blinded clinical trial to evaluate the safety and efficacy of a novel superelastic NiTi spinal rod in adolescent idiopathic scoliosis. In this work, in which the NiTi rod was mounted on the body spin through surgery, the results were acceptable [58].

**Figure 30:**
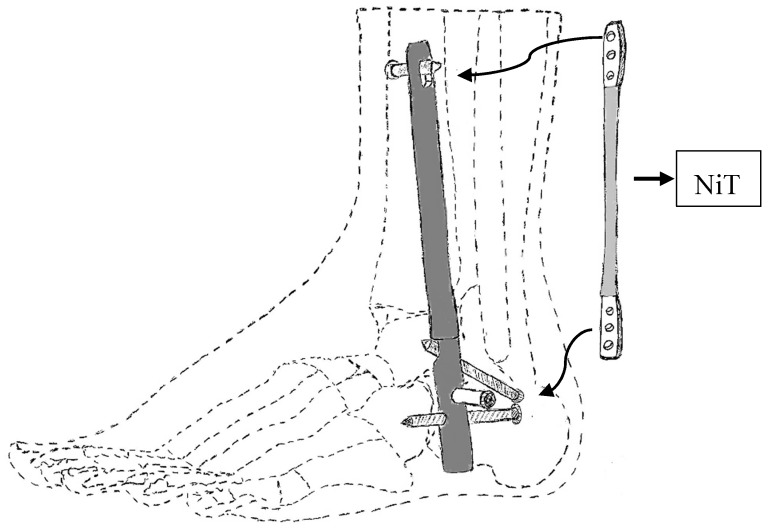
Arthrodesis device developed by Karnes et al. [59].

Arthrodesis, which is the artificial induction of joint ossification between two bones by surgery, is done to relieve intractable pain in a joint which cannot be managed by pain medication, splints, or other normally indicated treatments. Karnes et al. developed an arthrodesis device for generating and applying compression within joints (Figure 30). The device is capable of bringing bones in proximity to one another while generating a compressive load and maintaining the compressive load for a prolonged period of time until healing [59].

#### 2.2.5. SMAs in Rehabilitation Devices

##### Pittaccio et al.

**Figure 31:**
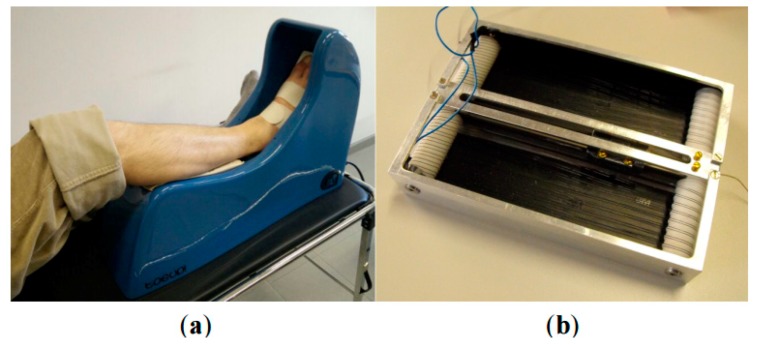
(**a**) The Toe-Up device for passive ankle mobilization of bedridden patients; (**b**) the shape memory alloy (SMA) actuator used to generate ankle dorsiflexion [32].

Pittaccio et al. have investigated the application of shape memory alloys (SMAs) for neurology and neuromuscular rehabilitation [32]. In this study, considering nonlinear properties of shape memory alloys (SMAs) such as pseudoelasticity (PE), shape memory effect (SME) and damping capacity, a couple of new medical devices have been designed and built to treat movement disorders such as dystonia or hyperkinesia. The first medical device which has been designed and built in this study is a kind of portable device, called a Toe-Up device, for a passive and aided exercise. As shown in Figure 31, the Toe-Up device has a leg rest which guides the foot on a moving plate that rotates up and down and creates plantar/ dorsiflexion in the ankle joint. 

The moving plate consists of a shape memory actuator and two pseudoelastic bias springs. The actuator is activated cyclically and can create 30 degrees dorsiflexion in the patient’s ankle, while the bias springs reset the position in the plantar flexion. The Toe-Up device has been utilized by seven pediatric patients affected by spastic tetraparesis for 30 min in a day for 14 continues days, and initial results indicate that Toe-Up device has an acceptable effect on these patients.

**Figure 32:**
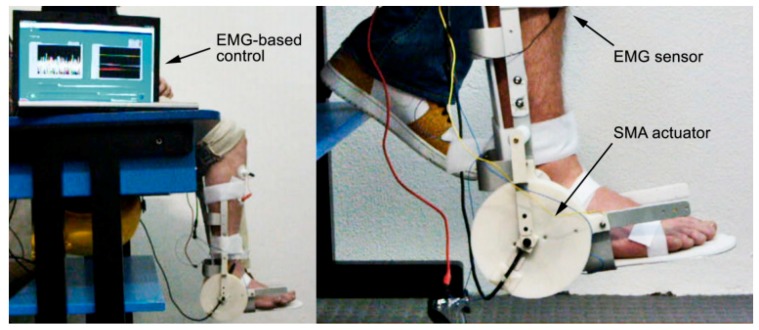
EMG-controlled SMA device for assisted ankle exercise [32].

Another rehabilitation device which has been designed and built in this study is a kind of active device that consists of an EMG sensor and an SMA actuator. Figure 32 shows the EMG-controlled SMA device for assisted ankle exercise. This device can be used for patients who have passed a series of therapies and can move their foot.

Designing a pseudoelastic hinge prototype by using an SMA spring is another example of a rehabilitation device that has been designed in this study. Figure 10 shows examples of pseudoelastic orthoses and the pseudoelastic hinge that has been used in them. 

##### Pittaccio et al.

**Figure 33:**
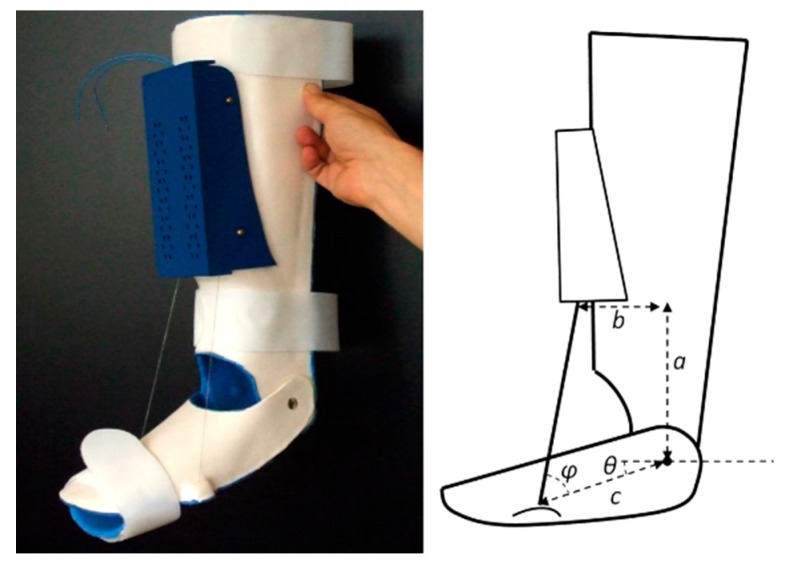
Implementation of an exerciser for the ankle joint with an SMA based actuator [60].

Pittaccio et al. [60] used an SMA based actuator in an AFO which consists of shank brace, foot brace, and an SMA actuator to design a new device for medical rehabilitation. Figure 33 shows the implementation of an exerciser for the ankle joint with an SMA based actuator. This device can create cyclic ankle motion including plantar/dorsiflexion by activation the SMA actuator. The SMA actuator includes two pseudoelastic bias springs and some Nitinol wires. By engaging the SMA actuator, the SMA wires are shortened to create dorsiflexion motions. Then, the two springs pull the ankle back to its original place for the next cycle.

**Figure 34:**
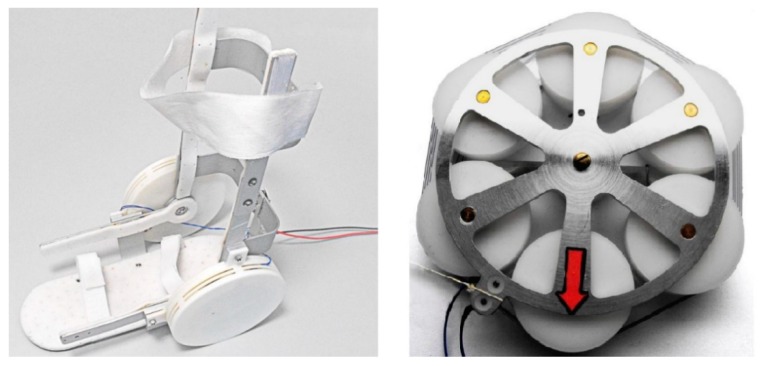
The application of a rotary SMA actuator in an AFO (in the left) as well as rotary SMA actuator (in the right) [60].

A rotary SMA actuator is another example of a rehabilitation device that has been designed in this study. Figure 34 shows the application of a rotary SMA actuator in an AFO (in the left) as well as a rotary SMA actuator (in the right). The rotary SMA actuator consists of two coils of wire traversed by the same current in the opposite direction.

##### Krishnan et al.

**Figure 35:**
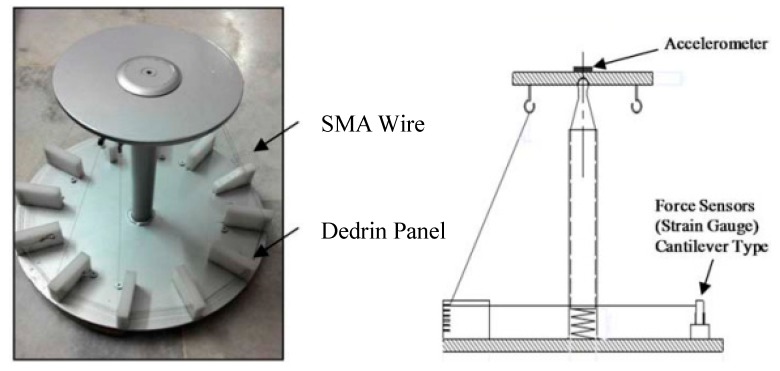
The SMA wires actuated Stewart platform rehabilitation device [61].

Krishnan et al. [61] utilized an SMA wire-actuated Stewart platform device to create a large range of motion for ankle rehabilitation therapy. Figure 35 illustrates how the SMA wires actuated Stewart platform rehabilitation device which has been designed and fabricated in this study. This device has two plates; the lower plate is fixed, but the upper plate can move due to the activation of SMA actuators. This device also has a three-axis accelerometer at the center of the upper plate to measure ankle angles. Six force sensors have been utilized to measure the force developed by the SMA actuators. 

**Figure 36:**
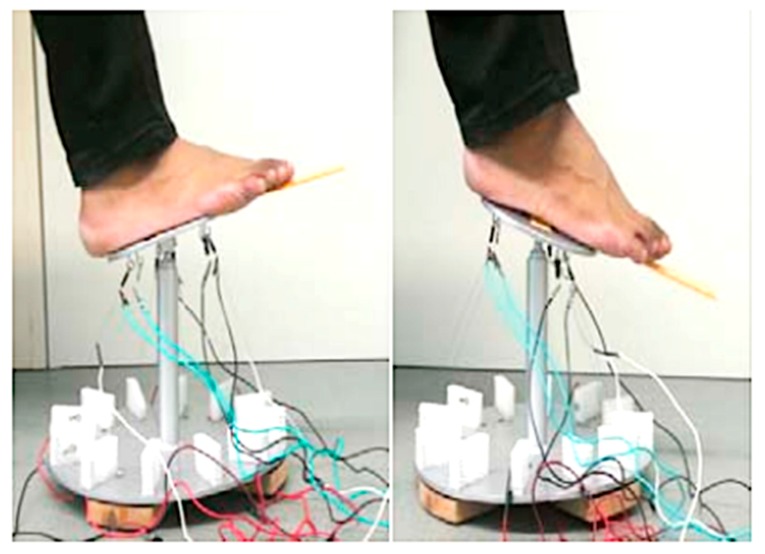
Experimental set up for the SMA wire actuated Stewart platform rehabilitation device [61].

The experimental set up for the SMA actuated Stewart platform rehabilitation device is shown in Figure 36. As can be found in Figure 36, this device can create +20/−20 degrees plantar/dorsiflexion and +19.87/−19.87 eversion/inversion in the ankle joint. These continuous passive motions (CPMs) help ankle rehabilitation therapy.

## 3. Discussion

### 3.1. Prosthetics

Regarding prosthetic hands, there are different parameters that severely restrict the range of use of the prosthesis. These parameters could be weight, DOF, the maximum applied force, or fabrication methods. These parameters can be classified for the abovementioned hands in Table 3.

Recent research has shown that shape memory alloys can be one of the most promising actuators. Low cost, noiseless operation, light weight, high power-mass ratio and simple actuation make SMAs interesting alloys to be utilized as actuators. However, there are several disadvantages compared to the current advance commercial devices, and to the authors’ knowledge there are no commercial prosthetic hands in the market using SMA as an actuator. Some of the problems that hinder commercialization can be summarized as follows.

- SMA wires need time to be cooled; hence the cooling rate greatly affects the efficiency of these type of actuation. There have been different methods like using forced cooling mechanisms or metallic heat sinks to improve the efficiency of the cooling. However, it should be noted that adding these items can influence the controlling issues as well as increasing weight and the need for space.
- One of the most difficulties of employing SMA as an actuator is its control complexity. Large temperature hysteresis, nonlinearities, lack of a reliable feedback signal due to changing SMA parameters are some of the parameters that make it hard to control this device. Many papers have been published on the SMA control methods that can address this issue and applying these methods on prosthesis hands can improve position and force control of these devices. For example, position control and stiffness control methods using strategies like nonlinear or adaptive controls, have been employed to control SMA-based actuators. Due to the lack of an accurate model for SMA based actuators and the presence of various uncertainties in the considered model, some researchers prefer to use non-model-based control methods [62,63]. The control method used in reference [62] is based on the several empirical rules while reference [63] is founded based on two-stage fuzzy controllers.
- Another interesting field of research that can be conducted for enhancing the hand prosthesis performance is to investigate the benefit of employing different kinds of SMA shapes like SMA springs or a bundle of wires. Also, a parametric study is needed on the length, diameter and the transformation temperature of SMA wires and their effect on the actuator performance.

### 3.2. Orthotics

So far, only one group has investigated the use of the dynamic knee actuating system for KAFO to improve abnormal ambulation of the knee caused by quadriceps weakness. Although the proposed KAFO has improved the stiffness profile when compared to the locked mode, still there is a large deviation between the dynamic mode and the normal behavior that needs to be addressed via further optimization of the KAFO. Also, the KAFO is super bulky due to the complicated actuation system, which makes it impossible for the patient to wear it. Possible future research on the topic of KAFOs will investigate: (i) improving and optimizing the mechanisms in order to make the KAFOs be more user-friendly, and have lower weight and volume; (ii) developing the KAFOs being able to accommodate more walking conditions, such as climbing stair, different walking speeds, and uneven grounds, and (iii) developing the passive KAFOs via taking advantage of superelasticity behavior of SMAs, such as the use of superelastic SMA based hinge, since it significantly reduces the complexity and volume of the device.

Different groups have investigated the use of AFOs to assist the patients in achieving a more natural gait and to prevent muscle atrophy. In these devices, the purpose was to increase the applied moment, provide normal ankle stiffness, enhance the range of ankle motion, and provide dorsiflexion at the end of the swing phase. In the work presented by Bhadane et al., the required characteristics of the SMA wires were calculated via conducting experiments on a hinged AFO without any wires and measuring the deviation from the healthy subjects. However, after mounting the appropriate SMA wires on the system, the modified device performance was not compared to the results of normal subjects. Taking advantage of this concept, Deberg et al. considered an actuation system to tune the walking by employing a pulley and SMA wires system. The ankle motion was significantly enhanced for the patient wearing the proposed AFO, which could be attributed to the flexibility of the SMA wires. However, in their work, the ankle stiffness profiles for different conditions were not plotted, which could be helpful for evaluation of the AFO stiffness. Also, the increase of applied moments on the patients compared to the healthy subject was not discussed. Possible future research on the SMA wire based AFOs will be: (i) finding a way to cover the wire and pulley systems; and (ii) tuning the ankle angle, ankle moment, and ankle stiffness simultaneously instead of focusing just on one outcome. Then, Mataee et al. [52] only implemented an adjustable superelastic tube and a hinge mechanism without using SMA wires any more. In their research, they only focused on the ankle stiffness of the normal subject. It is clear that they neglected the range of motion, the angle at the end of the cycle, and the moment on the ankle. Moreover, they only presented the finite element analysis results of their AFO, which were not validated in the work. However, the concept was of great value since the ankle stiffness of different walking conditions could be matched for normal condition and patient wearing AFO. It was obvious that the adjustable hinge was more successful in matching the stiffness of the subject walking in different conditions. Finally, Amerinatanzi et al. [54] took advantage of the superelastic behavior of SMA springs within the hinge. Their results demonstrated close to the normal range of motion, ankle stiffness, and applied moments, and dorsiflexion at the end of the cycle. This concept could be further optimized via considering different shapes and sizes of the SMA springs in order to completely match the characteristics of the normal subject.

Correcting deformities and abnormal postures of the body is another attractive field for utilizing SMA as an orthosis. In this application, much force must be applied to the bones or skeleton for a long time to slowly correct the abnormal shape. The property of SMA in preparing an almost constant force in a large range of deformation, exert and keep a large force on the abnormal part for a long period of time. This may result in a better treatment process with less pain and effort for the user. 

### 3.3. Rehabilitation

One of the disadvantages of SMA actuators is the low response time. In rehabilitation applications, there is no need for the users to move the disabled part fast, therefore the relatively slow movement of the rehabilitation device is acceptable. Further, the attractive properties of the SMA actuators as silent work, back drivability, and variable stiffness may result in developing the rehabilitation devices with more functionality in the future. 

## 4. Conclusions

The purpose of this paper is to review biomedical applications of NiTi SMAs in assistive and rehabilitation devices. Assistive devices are categorized to the prosthetic and orthotic devices including elbow, knee, ankle devices, and abnormal deformities correction. This review presented the NiTi assistive devices having a lightweight, silent operation, and high force and being able to mimic the motion of the proposed joint better than conventional devices. Results of this review indicated that there is a need to design assistive devices which can act as a good shock absorber and also tolerate large deformation. Regarding SMAs rehabilitation devices, it’s shown that future research should pay attention to developing more novel and practical rehabilitation devices by employing the unique properties of SMAs.

## Figures and Tables

**Table 1:** Clinical follow-up: ROM and spasticity (without EDGES) [29].

	Time	Sit	Stand	Walk	Relax	Ashworth
**A**	T_0_	60°	50°	65°	50°	3
	3 h	45°	45°	60°	50°	3
	24 h	45°	50°	60°	50°	3
	1 w	35°	40°	55°	40°	2
	1 w after	50°	60°	65°	60°	2
**B**	T_0_	70°	110°	100°	60°	2
	3 h	30°	60°	70°	50°	1+
	24 h	30°	30°	40°	30°	1
	1 w	25°	30°	40°	20°	1
	1 w after	65°	80°	95°	60°	2

**Table 2:** Information of some patients enrolled for the clinical study and the parameters taken into account in prescribing pseudoelastic hinges [33].

Subject	Age (Years)	Sex	Reaction Torque (N.mm)	Joint	AS	Group	ROM (°)
S04	12	F	364.34	Elbow	2	TP	80
S05	19	M	2062.76	Elbow	1	PT	126
S06	13	F	1741.22	Elbow	2	TP	124
S13	17	M	838.78	Elbow	1	TP	110
S14	17	M	−26.57	Elbow	2	TP	121

**Table 3:** Comparison of different prosthetic hands discussed in this paper.

Reference	Weight (kg)	DOF	Applicable or Gripping Force (N)	No. of Fingers	Note
The Hitachi Hand	4.49	12		4	
K. Laurentis and C. Mavroidis	1.36	20	6.67	5	one-step structure without the need for any further assembly
K. Andrianesis and A. Tzes	0.310	7	12	5	Additively manufactured has the shape and size of the average human hand
H. Taniguchi	-	-	10	5	prosthetic hand for children; Proposing a cooling system
Jae H. Lee et al	-	-		4(+1 fixed)	
